# Prevalence, Clinical, and Immunological Features of Familial Type 1 Diabetes Among Children and Adolescents: A Retrospective Study from Saudi Arabia

**DOI:** 10.3390/medicina61112066

**Published:** 2025-11-20

**Authors:** Raed Abutaleb, Saeed Yafei, Abdulrahman Hummadi, Yahia Solan, Abdullah Khawaji, Mohammed Hakami, Ali Jaber Alhagawy, Amer Al Ali, Morghema Adawi, Azizah Makrami, Fatima Bahsan, Molouk Mashhour, Lina Khardaly, Dalia Zahrani, Raga Johar, Nouf Algohani

**Affiliations:** 1 Adult Endocrinology and Diabetes Department, Jazan Endocrinology & Diabetes Center, Jazan Health Cluster, Ministry of Health, Jazan 82723, Saudi Arabia; 2 Endocrinology Department, Faculty of Medicine and Health Sciences, Taiz University, Taiz 6803, Yemen; 3 Pediatric Endocrinology, Jazan Endocrinology & Diabetes Center, Jazan Health Cluster, Ministry of Health, Jazan 82723, Saudi Arabia; 4 Nursing Department, Jazan Health Cluster, Ministry of Health, Jazan 86226, Saudi Arabia; 5 Family Medicine Department, Jazan Health Cluster, Ministry of Health, Jazan 86226, Saudi Arabia

**Keywords:** familial type 1 diabetes, children, autoimmune disease, Saudi Arabia, diabetic ketoacidosis, Arab countries

## Abstract

*Background and Objectives*: Familial type 1 diabetes (FT1D) represents a distinct subgroup of T1D potentially influenced by shared genetic and environmental factors. Data from Middle Eastern populations—where both T1D incidence and consanguinity are high—remain limited. This study aimed to determine the prevalence of FT1D and to compare the clinical, metabolic, and immunological features of FT1D with non-familial T1D (NFT1D) among children and adolescents in Saudi Arabia. *Materials and Methods*: A retrospective analytic study was conducted among 987 individuals diagnosed with T1D before 18 years of age and followed at the Jazan Endocrinology and Diabetes Center between 2015 and 2023. Participants were categorized as FT1D if they had at least one affected first-degree relative. Demographic, clinical, and biochemical data—including autoantibody profiles, associated autoimmune diseases, glycemic indices, and acute complications—were compared. Multivariate regression analyses were performed to assess independent associations after adjustment for age at diagnosis, sex, and parental consanguinity. *Results*: FT1D accounted for 19.5% of all T1D cases, with siblings being the most affected relatives (11.3%). FT1D patients were diagnosed at a younger age (8.2 ± 3.4 y vs. 9.3 ± 3.7 y; *p* = 0.001), had lower HbA1c (10.7 ± 1.5 vs. 12.0 ± 1.5; *p* < 0.001), less DKA at presentation (33.9% vs. 49.7%; *p* < 0.001), and fewer ICU admissions (13.5% vs. 20.8%; *p* = 0.023). In adjusted models, FT1D remained independently associated with lower odds of DKA (OR = 0.54, 95% CI 0.39–0.76, *p* < 0.001) and ICU admission (OR = 0.58, 95% CI 0.37–0.92, *p* = 0.019), and with higher odds of extra-pancreatic autoantibody positivity (OR = 1.78, 95% CI 1.21–2.61, *p* = 0.003) and anti-tissue transglutaminase antibodies (OR = 1.64, 95% CI 1.05–2.56, *p* = 0.031). *Conclusions*: FT1D constitutes a considerable proportion of pediatric T1D in Saudi Arabia and is characterized by earlier onset, milder metabolic decompensation at diagnosis, higher consanguinity, and higher likelihood of associated extra-pancreatic autoimmune diseases. Despite these differences, short-term glycemic outcomes remain similar to non-familial cases. These findings emphasize the need for family-based screening, genetic counseling, and early detection programs in high-risk populations.

## 1. Introduction

Type 1 diabetes (T1D) is a polygenic autoimmune disease characterized by destruction of insulin-producing pancreatic β-cells. The etiopathogenesis of T1D includes a complex interaction of genetic, environmental, and immunological factors [1]. Familial Type 1 diabetes (FT1D) refers to cases with at least one first-degree relative affected. A positive family history is one of the strongest risk factors, with first-degree relatives showing an 8–15-fold higher risk than the general population [2]. The risk varies according to the affected family member, being approximately 6–7% in siblings, 3–4% in offspring of affected mothers, and 6–9% in offspring of affected fathers [3]. This familial clustering suggests a strong genetic predisposition related to the human leukocyte antigen (HLA) and non-HLA loci. Patients with familial T1D carry the risk haplotype DR4-DQ8 more often than those with sporadic diabetes [2,4], indicating its role in familial aggregation. Although most T1D cases are non-familial (NFT1D), the prevalence of FT1D varied, with estimates ranging from 2% to 12.2% depending on the population investigated, observation period, and the study design [2,5,6,7,8]. Higher rates were reported in Finland, Sweden, Kuwait, and Saudi Arabia, while lower rates were observed in Asia and Latin America [1,5,9].

In type 1 diabetes, the T-cell-mediated autoimmune process is usually started early in life and marked by the production of β-cell-specific autoantibodies such as islet cell antibodies (ICA), glutamic acid carboxylase autoantibodies (GADA), insulin autoantibodies (IAA), and autoantibodies against zinc transporter 8 (ZnT8A), with risk increasing with the number of positive antibodies [10]. Familial and sporadic cases show similar overall autoantibody positivity, though differences in antibody frequency and patterns have been observed [2,4,11]. Because of their high genetic risk, first-degree relatives are key targets for preventive monitoring, as antibody number and age at seroconversion predict disease onset [12]. Moreover, shared immune dysregulation predisposes T1D patients to additional autoimmune diseases (AIDs), including autoimmune thyroiditis (up to 30%), celiac disease (4–9%), autoimmune gastritis (5–10%), Addison’s disease (0.5–1%), and vitiligo (2–10%) [13,14]. These AIDs may appear before, during, or after T1D diagnosis, with some studies reporting higher prevalence and earlier onset in familial T1D, influenced by age, sex, family history of AIDs, and type of familial relationship (parent–offspring versus sibling pairs) [6,14,15,16,17].

Most studies indicate that familial and non-familial T1D are distinct clinical entities at least at the time of diagnosis [6,7]. Turtinen et al. [4] showed that patients with FT1D were younger at diagnosis, whereas sporadic cases showed greater clinical and metabolic decompensation as measured by higher HbA1c, more severe weight loss, and a higher incidence of impaired consciousness. A meta-analysis by Usher-Smith et al. [18] found that children with FT1D have a 6-fold lower risk of DKA as the first manifestation of T1D. Furthermore, severe DKA and frequent admission to the intensive care units at diagnosis of T1D were more common in children without a family history of T1D [5,6].

Glycemic control in T1D patients is often complex and depends on different factors, including age, BMI, duration of diabetes, ethnicity, compliance with insulin therapy, type of insulin therapy, access to healthcare facilities, family support, eating behavior, and psychological effects of living with a chronic disease [19]. Glycated hemoglobin (HbA1c) and ambulatory glucose profile (AGP) are the usual determinants of glycemic control in addition to the annual screening for long-term complications. Studies regarding the long-term outcomes among patients with FT1D are controversial. Some studies reported similar HbA1c levels and ketoacidosis rates between familial and sporadic cases over the long-term follow-up period [6,20,21]. However, other studies reported higher mean HbA1c values and diabetic ketoacidosis rates in familial cases throughout the long-term follow-up [7,22].

Saudi Arabia ranks among the top 10 countries worldwide in both incidence and prevalence of childhood T1DM. The disease burden and prevalence have escalated rapidly over recent decades. The incidence had increased from 18.5 per 100,000 to 36.00 per 100,000 children at or below the age of 14, with urban regions reporting the highest rates [23]. The genetic study in a Saudi cohort by Eltayeb-Elsheikh et al. [24] confirmed the central role of certain HLA haplotypes, particularly HLA-DR3, DR4, and T1D. That study indicated a higher frequency of HLA-DRB103 and HLA-DRB104 alleles in Saudi children with T1D, similar to global findings. Unique haplotypes, including the HLA-DQB1*02:01, have also been reported, suggesting possible population-specific risk profiles [24]. Associations with non-HLA genes such as INS and PTPN22 have also been reported, though less consistently than those for HLA loci [25]. The most recent VISION-T1D program [26] represents a landmark national initiative, employing advanced genetic screening—including HLA typing, islet autoantibody profiling, and genetic risk scoring (GRS2)—in children with first-degree relatives affected by T1DM. Such genetic initiatives hold promise for refining early detection, risk stratification, and eventually prevention strategies tailored to the Saudi population.

Familial T1D studies were conducted mostly in European countries; however, only a few studies were conducted in Arab countries. Upon reviewing the available literature, only one population-based study was conducted in Kuwait, in addition to two small cross-sectional studies from Saudi Arabia and Qatar [4,5,27]. In a community with high prevalence and incidence of T1D among children and adolescents, it is of great importance to understand the epidemiological and clinical characteristics and genetic predispositions of different phenotypes of T1D. The current study is trying to answer the question about the phenotypic characteristics of familial T1D. Thus, we conducted this study to investigate the prevalence of FT1D children and adolescents in Saudi Arabia with a particular focus on comorbidities, clinical characteristics, and immunological profiles compared with non-familial T1D.

## 2. Materials and Methods

### 2.1. Study Design and Population

**Figure 1:**
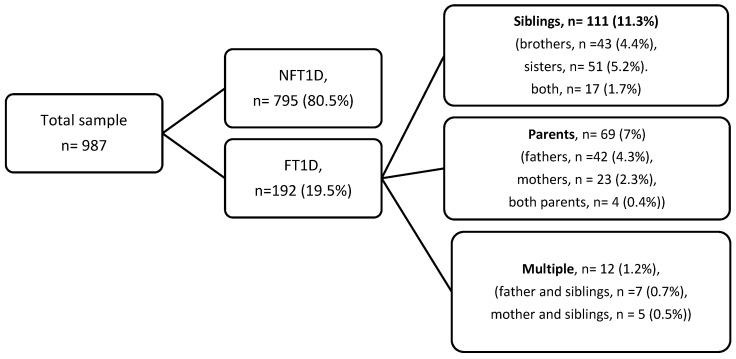
Flow diagram of participant selection and distribution of familial and non-familial Type 1 diabetes subgroups (percentages shown).

This is a retrospective observational analytic study comparing children and adolescents with familial and non-familial type 1 diabetes. Retrospective data were collected from the electronic medical records to include children and adolescents with T1D who were diagnosed over the period from January 2015 to December 2023. The total sample included 987 children and adolescents diagnosed with T1D below the age of 18 years (Figure 1). The FT1D group included participants with T1D who have any parents or siblings with type 1 diabetes. The NFT1D group included children and adolescents with no family history of T1D in any of their first-degree relatives.

All children and adolescents with type 1 diabetes who were diagnosed at or below the age of 18 years and were on regular follow-up at the pediatric and adolescent clinics at Jazan Endocrinology and Diabetes Center, Saudi Arabia. We excluded children with diabetes diagnosed before 6 months or after 18 years of age, those diagnosed before 2015, and patients with a diabetes duration < 1 year. Participants were included if complete data were available for the date of diagnosis, clinical presentation at diagnosis, admission records, C-peptide levels, autoantibody status, HbA1c at diagnosis, and at least one year of follow-up. Patients with unconfirmed T1D and those with any type of diabetes other than T1D were excluded. T1D was confirmed according to the International Society for Pediatric and Adolescent Diabetes (ISPAD) guidelines and categorized according to their clinical profiling, C-peptide level, and T1D autoantibodies [28].

### 2.2. Clinical and Laboratory Variables

Socio-demographic and T1D-related data, including age at diagnosis, duration of diabetes, number and degree of affected first-degree relatives, parental consanguinity, socioeconomic standards, age of T1D diagnosis in the relatives, and presence of other autoimmune diseases. A specific checklist was developed for this study to be filled out by trained investigators for each patient. The checklist includes patient sociodemographic and clinical data, in addition to enquiries about T1D in the first-degree relatives.

Clinical, anthropometric, and biochemical data were retrieved. Medical information regarding the onset of T1D, clinical presentation with DKA at diagnosis, venous blood gases records at diagnosis, HbA1c at first presentation, presentation with DKA, and history of admissions to the ICU at diagnosis were retrieved. Body mass index (BMI) (kg/m^2^) was calculated from weight (kg) and height (m) and adjusted for age and sex.

Glycemic control was assessed by mean HbA1c levels within the last year before inclusion in the study by averaging the values of HbA1c for the year. Data on severe hypoglycemia events and/or admission with DKA within one year before inclusion were retrieved from the electronic medical records and analyzed. DKA was defined as plasma glucose ≥ 200 mg/dL with venous pH ≤ 7.30 and bicarbonate < 15 mEq/L. Severe hypoglycemic events were considered if the patient had low blood glucose < 70 mg/dL, they developed coma and/or seizures, or were treated with glucagon injection or intravenous glucose infusion. Data regarding insulin therapy were also collected, including insulin regimen (basal–bolus, premixed, or pump therapy) and use of carbohydrate counting for dose adjustment.

### 2.3. Autoimmune Markers

We screened the medical records for pancreatic autoantibodies, which were usually requested either at the first presentation or within the first three months of diagnosis, to include glutamic acid decarboxylase antibody GADA, islet cell antibody ICA, and anti-insulin antibody IAA. For the assessment of associated autoimmune diseases, we checked for a history of overt hypo- or hyperthyroidism, celiac disease, and vitiligo. Each file was screened for thyroid function tests, thyroid peroxidase antibody (TPO), and anti-tissue transglutaminase IgA (TTG IgA) antibody regardless of history of overt disease. Endoscopic and pathologic reports for celiac disease were reviewed to confirm biopsy-proven celiac disease.

### 2.4. Ethical Consideration

This study was performed under the Declaration of Helsinki and granted ethical approval from the Jazan Health Cluster Ethics Committee, Saudi Arabia, Approval No. H-10-Z-141, 25132, 2025. Parental consent and patient assent were obtained at the time of clinical care in accordance with institutional policy.

### 2.5. Statistical Analysis

Statistical analyses of the data were conducted using SPSS Version 26 (IBM, Armonk, NY, USA). Sociodemographic and clinical characteristics were described by descriptive analysis. Count and percentage values were calculated for categorical variables. Normality of continuous variables was assessed visually and by the Shapiro–Wilk test. Variables with a normal distribution were analyzed using independent *t*-tests, whereas the Mann–Whitney U test was applied for non-normally distributed variables. Categorical variables were analyzed using the Chi-square test, and Fisher’s exact test was applied for variables with low expected frequencies. Logistic and linear regression models were constructed to identify independent associations between family history of T1D and key clinical outcomes (DKA at diagnosis, C-peptide level, HbA1c at inclusion, and autoimmune comorbidities). The models included age at diagnosis, sex, and parental consanguinity as covariates. Bonferroni’s correction for multiple comparisons was not applied due to its overly conservative nature. The level of significance was determined at *p* < 0.05.

## 3. Results

### 3.1. Prevalence, Sex, and Age Distribution

This study included 987 children and adolescents diagnosed with T1D below the age of 18 years. T1D females accounted for 56.6% of the total sample. We identified 192 participants (19.5%) who have one or more first-degree relatives with T1D (Figure 1). Of the FT1D, 111 participants (11.3%) had at least one sibling with T1D. Of the sample, 42 participants had a father with T1D (4.3%), and 23 participants (2.3%) had a mother with T1D. Twelve participants had at least one sibling and one parent with type 1 diabetes (1.2%). Consanguineous marriage among the FT1D parents was more prevalent in the FT1D group (47.9%) compared to the non-familial group (37.4%) (*p* = 0.007). Both sexes were equally distributed in both groups; 53.1% of the FT1D group were females vs. 54.5% of the NFT1D group (*p =* 0.74). Both males and females had equal occurrence of a first-degree relative with T1D, 19.9% of the boys and 19.1% of the girls. At diagnosis, the mean age in the FT1D group was lower (8.2 ± 3.4 years) than in the NFT1D group (9.3 ± 3.7 years) (*p* = 0.001). At inclusion, the mean duration of diabetes was higher in the familial group, 5.4 ± 2.8 years vs. 4.6 ± 2.7 years in the non-familial group (*p* = 0.001). Other sociodemographic and clinical features of the participants are provided in Table 1.

### 3.2. Clinical Presentation at Diagnosis

At diagnosis, DKA was documented in 47.7% of the total sample. DKA was less frequent in the FT1D compared to the NFT1D cases, 33.9% vs. 49.7%, (*p* < 0.001). The FT1D group had a slightly lower level of blood glucose at disease onset, 431 vs. 457 mg/dL (*p* < 0.001). Admission to the ICU at diagnosis was documented in 13.5% of FT1D vs. 20.8% of the non-familial group (*p* = 0.023). The initial insulin doses were lower in the familial group compared with non-familial cases during the first year, 0.6 vs. 0.8 IU/kg/day, (*p* < 0.001). Familial T1D had relatively lower HbA1c at diagnosis, 10.9% ± 1.5 vs. 12% ± 1.5 in the NFT1D (*p* < 0.001), and higher C peptide level, 0.11 (IQR = 0.08–0.15) vs. 0.07 (IQR = 0.05–0.10), *p* < 0.001. However, at inclusion, patients with familial diabetes had similar HbA1c compared to the non-familial cases, 8.7% ± 2.1 vs. 8.5% ± 2, (*p* = 0.28).

The overall incidence of DKA events and severe hypoglycemia within the last year before inclusion in this study was comparable in both groups (Table 1). About 10% of the total sample were on insulin pump therapy, and 28% practiced carb counting in insulin dose calculation.

### 3.3. Autoimmune Markers

Overall, about two-thirds of the total sample had at least one positive anti-pancreatic autoantibody (66.5%). GADA was positive in 61.3% of the total sample, followed by IAA (40.2%) and ICA (31.6%) antibodies. GADA, ICA, IAA, and the overall anti-pancreatic antibody proportions were comparable in both groups (Table 2).

Regarding the associated autoimmune diseases, approximately 19.8% of the total sample tested positive for at least one extra-pancreatic autoantibody, 26% in the FT1D vs. 17.2% in NFT1D, (*p* = 0.005). TPO antibody was positive in 13.7% and TTG-IgA antibody in 12.3% of the total sample. FT1D had 16.7% seropositive TTG-IgA antibody, compared to 11.2% of the NFT1D, (*p* = 0.038). Overt celiac disease, documented by endoscopy or a 10 times titer of TTG IgA antibody, was the most prevalent disease (9.2%), followed by hypothyroidism (6.7%), hyperthyroidism (1.6%), and vitiligo (0.9%). Celiac disease was more prevalent in the FT1D group, 13%, compared to 8.3% in NFT1D, (*p* = 0.043). Thyroid peroxidase antibody, hypothyroidism, hyperthyroidism, and vitiligo were more frequent in the FT1D; however, the differences were not significant for all (Table 2).

### 3.4. Multivariate Analysis

Multivariate analysis was conducted to identify independent associations between familial type 1 diabetes (FT1D) and key clinical and immunological outcomes after adjustment for age at diagnosis, sex, and parental consanguinity. As shown in Table 3, children, and adolescents with FT1D had significantly lower odds of presenting with diabetic ketoacidosis (DKA) at diagnosis (OR = 0.54, 95% CI: 0.39–0.76, *p* < 0.001) and reduced likelihood of intensive care unit admission at presentation (OR = 0.58, 95% CI: 0.37–0.92, *p* = 0.019) compared with non-familial cases. Conversely, FT1D was independently associated with a higher probability of testing positive for extra-pancreatic autoantibodies (OR = 1.78, 95% CI: 1.21–2.61, *p* = 0.003) and anti-tissue transglutaminase antibodies (OR = 1.64, 95% CI: 1.05–2.56, *p* = 0.031), indicating an increased susceptibility to associated autoimmune comorbidities, particularly celiac disease.

In the linear regression model (Table 3), FT1D status was a significant predictor of multiple continuous variables: patients with FT1D were diagnosed approximately one year earlier (*B* = −1.00, 95% CI: −1.57 to −0.43, *p* < 0.001), had lower blood glucose at presentation (*B* = −22.6 mg/dL, 95% CI: −39.5 to −12.2, *p* < 0.001), higher C-peptide levels (*B* = 0.053, 95% CI: 0.042–0.064, *p* < 0.001), lower HbA1c at diagnosis (*B* = −1.01, 95% CI: −1.25 to −0.77, *p* < 0.001), and lower initial insulin dose requirements (*B* = −0.19, 95% CI: −0.22 to −0.17, *p* < 0.001). Together, these findings confirm that FT1D remains independently associated with earlier disease onset, milder metabolic decompensation at diagnosis, and greater co-occurrence of autoimmune markers, even after adjusting for key confounders.

## 4. Discussion

This study was carried out in Jazan, a densely populated area in southwestern Saudi Arabia. The most significant finding of this study is that the prevalence of FT1D was 19.5%, with siblings being the most affected first-degree relatives. Interestingly, children and adolescents with FT1D presented at a younger age, experienced less severe metabolic decompensation at diagnosis, showed higher parental consanguinity, and exhibited numerically higher rates of certain autoimmune comorbidities (thyroid disease, celiac disease) while overall pancreatic antibody positivity was broadly similar between both groups. Familial aggregation of T1D is a well-established phenomenon that has been studied in different regions of the world; however, its prevalence among the first-degree relatives showed inconsistent results.

Given the genetic and cultural homogeneity of the Saudi population, along with the exceptionally high incidence of T1D, investigating the familial distribution and characteristics of the disease represents both a suitable and an important healthcare priority [29]. In this cohort, a large sample of children and adolescents was included, and we also included the parental history of T1D, not only the siblings. The overall prevalence of FT1D in this cohort, and the prevalence among the siblings (11.3%), were not so different from what was reported in the other Gulf countries, which shared similar genetic backgrounds, weather, living conditions, and economy [5,9,27,30]. An early report from Saudi Arabia indicated that 15.9% of children with T1D had at least one sibling also affected [9]. Similarly, a retrospective study from Qatar reported FT1D prevalence in 14.6% of the T1D sample, with male predominance and an earlier age of onset in the familial group [27]. Both studies, however, were limited by small sample sizes and inclusion criteria restricted to sib-pair cases of T1D, excluding family history in the parents, which might underestimate the true prevalence rate of FT1D. More recently, a study from Kuwait [5] reported a prevalence of FT1D of 11.2% in children below 12 years of age. Although the last study was population-based with a large sample size, it did not include children and adolescents above the age of 12 years, so it could also underestimate the true prevalence of FT1D [5]. Another retrospective study from Oman showed FT1D prevalence in 15.3% of children with a similar clinical presentation of both groups [30].

However, reports from Western countries are much lower than our findings. A recent population-based report from the Diabetes Prospective Follow-up Registry (DPV) in 2021 found that FT1D was prevalent in 6.6% of T1D patients below the age of 20 in Germany, Austria, Switzerland, and Luxembourg [6]. Other reports from the Finnish Pediatric Diabetes register had reported FT1D at a rate of 12.2% in 2012 and 10.4% in 2019 [2,4], however, an earlier population-based study from Finland reported a higher prevalence of 15.1% in first-degree relatives [11]. Other reports from Sweden and Ireland showed rates of 10.3% and 10.2, respectively [31,32].

This relatively high prevalence of FT1D in Saudi Arabia and other Arab countries might be related in part to the shared genetic background and the high rates of consanguinity. Consanguineous marriage is very popular in the Saudi community, where about 50% of Saudi marriages are consanguineous [33]. In the current study, the consanguineous marriage among the FT1D parents was prevalent in 47.9% of the parents, mostly among the first-cousin marriages. Albishi et al. [34] showed that children of the first cousin parental marriage are at high risk of T1D. This risk became more obvious in families with a family history of T1D and consanguinity. A genetic study of T1D in Saudi children by Manan et al. [25] found that a different combination of alleles at the DRB1 and DQB1 loci is a critical determinant for T1D risk. The DQB1*0201/DQB1*0302 had the highest association with T1DM in Saudi children. However, genetics and consanguinity are not the sole factors that could explain this high prevalence of FT1D in Saudi children and adolescents. Different variables could be implicated, including viral infections, nutritional factors, lifestyle changes, breastfeeding practices, and exposure to unidentified risk factors that clustered in certain families [35].

Interestingly, our study demonstrated that participants with FT1D were most frequently observed to have a sibling with T1D, 11.3% of the patients have at least one affected sibling, and 7% have at least one affected parent; 4.3% have an affected father and 2.3% have a mother with T1D. These findings of high transmission among siblings align with some reports but contrast with others. For instance, the DPV documented that 3.2% of FT1D cases had an affected parent and 3.6% had an affected sibling [6]. Earlier research from the United States, Finland, and Germany suggested that T1D was more often transmitted through the father, followed by the mother and siblings [2,4,36]. Similar to our findings, studies from similar communities in Saudi Arabia and Kuwait showed that T1D was more likely to be prevalent among siblings [5,9]. This pattern of higher sibling occurrence than parent–offspring transmission of T1D could be influenced by the shared genetic determinants and common environmental exposures during early life.

Regarding the age of first presentation of T1D in the familial group, it was earlier than in the non-familial group, 8.2 ± 3.4 vs. 9.3 ± 3.7 years, respectively, (*p* = 0.001). The earlier presentation of T1D in FT1D was confirmed after adjustment for age, sex, and consanguinity in the linear regression (*B* = −1.00 years, 95% CI −1.57 to −0.43; *p* < 0.001). Similar to our results, the earlier onset of T1D in the FT1D group was reported in Finland (6.9 vs. 8.5 years) [11] and in the DPV (7.9 vs. 9.7 years) [6]. In contrast, there have been multiple reports that showed no difference in age of onset comparing FT1D and NFT1D [4,5,9,15,35,37]. In Saudi Arabia and Kuwait, T1D children with or without affected siblings usually presented at the age of 8 years [5,9], while in Qatar, FT1D often appeared earlier in childhood [27]. In terms of gender, we found no gender differences in FT1D presentation, which is consistent with most regional and international reports [4,5,6,9]

Whether familial or sporadic, T1D patients usually share the hallmark clinical symptoms of hyperglycemia, which include polyuria, polydipsia, weight loss, and ketoacidosis. In this cohort, participants with FT1D had lower HbA1c (*B* = −1.01, 95% CI −1.25 to −0.77; *p* < 0.001) and higher C-peptide levels (*B* = 0.053, *p* < 0.001) at diagnosis than those with NFT1D. The prevalence of diabetic ketoacidosis at diagnosis in our cohort was lower in the familial group, 33.9% vs. 49.7% in the NFT1D group, *p* < 0.001. Logistic regression analysis demonstrated that FT1D was associated with significantly lower odds of presenting with DKA at diagnosis (OR = 0.54, *p* < 0.001), and of ICU admission at presentation (OR = 0.58, *p* = 0.019). Similar findings have been reported in several studies [4,6,9], most likely reflecting earlier recognition of symptoms within families already familiar with diabetes. Nonetheless, some studies suggest a biological difference, with paternal transmission associated with a more severe presentation than maternal transmission [4]. Interestingly, the proportion of DKA in the familial group was highly elevated when compared to the international reports, such as the DPV (11.9%) [6] and the Finnish Registry (7.8%) [4]. This discrepancy underscores that, even among families with prior exposure to type 1 diabetes and within a healthcare system offering readily accessible diabetes services, the occurrence of DKA continues to represent a substantial and unacceptable burden on the population [38].

Consistent with previous reports, our findings showed that the frequencies of GADA, ICA, IAA, and the overall pancreatic autoantibodies were comparable between familial and non-familial T1D groups. Most studies in the literature similarly describe no significant differences in pancreatic autoantibody profiles, supporting the notion of shared immunopathogenic mechanisms underlying both FT1D and NFT1D [2,11,39]. However, some discrepancies exist. For instance, Lebenthal et al. [15] and Turtinen et al. [4] reported higher IAA positivity in familial cases, whereas a study from Kuwait found lower odds of GADA and IAA positivity in FT1D children, particularly those with affected siblings [5]. Overall, the absence of major differences in pancreatic autoantibody patterns between familial and non-familial cases may reflect shared HLA-linked genetic susceptibility within this population. These genetic factors might induce similar pancreatic autoimmune processes in both groups, beginning during the prediabetic phase, irrespective of family history [11].

In individuals with T1D, the coexistence of additional autoimmune diseases is common and often correlates with distinct clinical and immunological characteristics. Consequently, routine screening for autoimmune antibodies is essential to identify subclinical disease and prevent complications arising from unrecognized comorbid autoimmunity. Previous research has shown variability in reported prevalence rates, largely due to differences in study design—some investigations assessed AIDs only at the time of T1D diagnosis, whereas others followed patients longitudinally, leading to variations across populations [5,6,16]. Data from the DPV longitudinal pediatric study demonstrated that AIDs were present in 16.7% of FT1D and 13.6% of sporadic cases, with a much higher frequency in the sib-pairs than in the parent-offspring subgroup [6]. In adults, the prevalence appears to be even greater; Milluzzo et al. [16] reported both an earlier onset and a higher rate of coexisting AIDs in familial T1D (29.8%) compared with sporadic cases (18.4%). In the present study, FT1D was independently associated with a higher likelihood of positivity for extra-pancreatic autoantibodies (OR = 1.78, *p* = 0.003) and anti-tissue transglutaminase antibodies (OR = 1.64, *p* = 0.031), indicating increased susceptibility to coexisting autoimmune disorders—particularly celiac disease. Generally, the high association between T1D and celiac disease in Saudi children was reported previously [40]. In the current study, celiac disease was more prevalent in FT1D compared to non-familial T1D (13% vs. 8.3%, *p* = 0.043). The DPV reported a notable excess of celiac disease in familial versus sporadic T1D (6.2% in FT1D vs. 4.2% in sporadic cases) [6]. Hypothyroidism and hyperthyroidism were also more prevalent in the FT1D group; however, the difference was not significant.

Regarding the glycemic control and acute complications, both familial and non-familial T1D groups showed comparable rates of acute complications (Table 1). At the inclusion in this study (a median duration of 4 years (IQR = 2–7 years), the means of HbA1c, and the rates of DKA and severe hypoglycemia events were similar in both groups, indicating comparable short-term metabolic risks regardless of family history. These findings signify that FT1D had a heterogenous phenotype where the peculiar initial presentation with less severe decompensation does not always translate into long-term outcome differences. A similar finding was reported by O’Leary et al. [21], where both groups had comparable HbA1c and insulin doses at 1-year follow-up. Reddy et al. [20] found that HbA1c at diagnosis and over 5 years were similar in familial and sporadic T1D. In contrast, a retrospective study by Lebenthal et al. [7] reported that FT1D patients have higher mean HbA1c values and a higher incidence of DKA events than the sporadic cases throughout the long follow-up period. However, severe hypoglycemia and microvascular complications were similar in both groups in the same study [7]. Another study by Vakharia et al. [22] found that family history of diabetes was associated with more frequent DKA in pediatric patients with established T1D, regardless of the type of familial diabetes or the first-degree relative status.

The comparable rates of severe hypoglycemia and glycemic control in our cohort suggest that, once insulin therapy is initiated, metabolic outcomes are more strongly influenced by behavioral, educational, and technological factors than by family history itself. The relatively low proportion of insulin pump users in both groups (~10%) highlights an opportunity to expand access to advanced diabetes technologies.

In summary, this study explored the characteristics of familial type 1 diabetes compared with nonfamilial cases within one of the world’s highest-incidence T1D populations. Taken together, this study showed that familial T1D differs from non-familial disease in meaningful ways: FT1D is less common than sporadic disease but demonstrates a marked clustering among first-degree relatives, reinforcing the relevance of targeted screening and monitoring strategies for earlier recognition and intervention in families affected by T1D. Increased awareness and structured education for first-degree relatives could help reduce the risk of severe presentations such as DKA at diagnosis. Furthermore, given the higher prevalence of associated autoimmune diseases observed in the familial group, these findings support the implementation of enhanced autoimmune screening programs among relatives of T1D patients, including periodic assessment for thyroid and celiac autoimmunity. Family-centered screening and education programs may improve early detection and long-term outcomes in high-risk populations.

Despite the observational nature of this study, our findings highlight that family history may influence the clinical presentation and autoimmune profile in T1D, but further prospective studies are needed to explore underlying mechanisms. A major strength of this study lies in its large sample with efficient medical records, which ensures comprehensive data collection from a well-defined population. The inclusion of a high-incidence region adds global relevance and highlights regional differences in familial clustering. The use of digitalized medical records enhanced data accuracy and minimized extraction bias. Furthermore, the study provides an important foundation for understanding familial aggregation of T1D in a population with distinct genetic and demographic characteristics.

Despite its strengths, several limitations should be acknowledged. The retrospective design limited the ability to assess long-term clinical outcomes or disease progression. Some potentially relevant data may be missed from the records. The study was limited to a single center, which may introduce selection bias and limit the generalizability of the findings. Multicenter studies are warranted to validate these results in other regions. Moreover, the absence of genetic data or DNA banking limited exploration of underlying hereditary mechanisms. Future research should adopt multicenter, longitudinal designs with integrated genetic and environmental data to clarify the hereditary and immunological pathways driving familial T1D.

## 5. Conclusions

This study highlights that familial type 1 diabetes (FT1D) accounts for a considerable proportion of children and adolescents with T1D in Saudi Arabia. FT1D patients presented at a younger age with milder metabolic decompensation at diagnosis as evidenced by lower HbA1c, higher C-peptide levels, and lower rates of DKA and ICU admission. However, follow-up findings revealed similar glycemic control, hypoglycemia, and DKA rates between familial and non-familial groups. FT1D therefore represents a group with distinct presentation features rather than a sustained phenotype, emphasizing the need for early recognition, family-centered education, and proactive screening to improve outcomes in high-risk families. The observed higher prevalence of celiac disease and extra-pancreatic autoantibodies further highlights the need for periodic autoimmune screening and family-based education in populations with high T1D incidence.

## Figures and Tables

**Table 1:** Demographic, clinical, and metabolic data grouped by positive family history of T1D in first-degree relatives.

	Overall *n* = 987	FT1D (*n* = 192)	NFT1D (*n* = 795)	*p* *	Adjusted *p* **
Males, *n* (%)	452 (45.8)	90 (46.9)	362 (45.5)	0.74	0.58
Females, *n* (%)	535 (54.2)	102 (53.1)	433 (54.5)		
Consanguinity, *n* (%)	389 (39.4)	92 (47.9)	297 (37.4)	0.007	0.011
Age at diagnosis, years, mean (SD)	9.1 (3.6)	8.2 (3.4)	9.3 (3.7)	0.001	<0.001
**Clinical and metabolic data at diagnosis**					
Serum blood glucose, mg/dL	452 (87)	431 (84)	457 (87)	<0.001	0.001
C-peptide level, nmol/liter, median (IQR)	0.07 (0.05–0.11)	0.11 (0.08–0.15)	0.07 (0.05–0.10)	<0.001	<0.001
DKA at diagnosis, *n* (%)	460 (46.6)	65 (33.9)	395 (49.7)	<0.001	<0.001
ICU admission, *n* (%)	191 (19.4)	26 (13.5)	165 (20.8)	0.023	0.019
HbA1c, mean (SD)	11.8 (1.6)	10.9 (1.5)	12 (1.5)	<0.001	<0.001
Initial Insulin dose, U/kg/d	0.8 (0.2)	0.6 (0.1)	0.8 (0.2)	<0.001	<0.001
**Clinical and metabolic data at inclusion**					
Age at inclusion, years, mean (SD)	13.8 (3.5)	13.5 (3.2)	13.8 (3.5)	0.26	0.26
Duration, years, mean (SD)	4.7 (2.7)	5.3 (2.8)	4.6 (2.7)	0.001	0.001
HbA1c at inclusion, mean (SD)	8.6 (2)	8.7 (2.1)	8.5 (2)	0.28	0.44
DKA history in the last year, *n* (%)	69 (7)	16 (80.2)	53 (6.7)	0.42	0.6
Severe hypoglycemia in the last year, *n* (%)	181 (18.3)	41 (21.4)	140 (17.6)	0.23	0.32
**Management modality**					
Carb counting, *n* (%)	328 (33.2)	56 (29.2)	272 (34.2)	0.20	0.12
Insulin pump users, *n* (%)	87 (8.8)	19 (9.9)	68 (8.7)	0.86	
Basal bolus insulin, *n* (%)	841 (85.2)	162 (84.4)	679 (85.4)		0.56
Pre-mixed insulin, *n* (%)	58 (5.9)	11 (5.7)	47 (5.9)		

Data are presented as number (*n*), mean, and standard deviation (SD). p *: *p* value indicates significance at the 0.05 level. ** Adjusted *p* in linear and logistic regression models after adjustment for age at diagnosis, gender, and consanguinity.

**Table 2:** Autoantibody profile and associated autoimmune diseases in familial type 1 diabetes (FT1D) and non-familial type 1 diabetes (NFT1D).

	Overall *n* = 987	FT1D (*n* = 192)	NFT1D (*n* = 795)	*p* *	Adjusted *p* **
Pancreatic autoantibody positivity rate, *n* (%)	656 (66.5)	134 (69.8)	522 (65.7)	0.28	0.25
Glutamic acid decarboxylase antibody (GADA), *n* (%)	605 (61.3)	112 (58.3)	493 (62)	0.35	0.36
Insulin auto-antibody (IAA), *n* (%)	397 (40.2)	83 (43.2)	314 (39.5)	0.34	0.27
Anti-islet cell antibody (ICA), *n* (%)	312 (31.6)	51 (26.6)	261 (32.8)	0.09	0.12
Overall extra-pancreatic autoantibody positivity, *n* (%)	187 (18.9)	50 (26)	137 (17.2)	0.005	0.003
Thyroid peroxidase antibody, *n* (%)	135 (13.7)	34 (17.7)	101 (12.7)	0.07	0.058
Anti-tissue transglutaminase antibody, *n* (%)	121 (12.3)	32 (16.7)	89 (11.2)	0.038	0.031
Hypothyroidism, *n* (%)	66 (6.7)	17 (8.9)	49 (6.2)	0.18	0.11
Hyperthyroidism, *n* (%)	19 (1.9)	5 (2.6)	14 (1.8)	0.45 ^b^	0.44
Celiac disease, *n* (%)	91 (9.2)	25 (13)	66 (8.3)	0.043	0.045
Vitiligo, *n* (%)	9 (0.9)	3 (1.6%)	6 (0.8)	0.29 ^b^	0.29

p *: *p* value was calculated for both familial and non-familial groups, significant at the 0.05 level. ** Adjusted *p* in linear and logistic regression after adjustment for age at diagnosis, gender, and consanguinity. ^b^: Fisher’s exact test was used for comparison.

**Table 3:** Associations between familial type 1 diabetes (FT1D) and clinical, metabolic, and immunological outcomes compared with non-familial type 1 diabetes (NFT1D).

Logistic Regression Model	*β* = Odds Ratios, 95% Confidence Interval	*p*
DKA at diagnosis	0.540 (0.387, 0.755)	<0.001
ICU admission at diagnosis	0.583 (0.371, 0.916)	0.019
Overall extra-pancreatic autoantibodies	1.778 (1.1214, 2.605)	0.003
Anti-tissue transglutaminase antibody	1.637 (1.046, 2.563	0.031
**Linear Regression Model**	***B* = Unstandardized Coefficient, 95% CI**	
Age at diagnosis, years *	−1 (−1.573, −0.428)	<0.001
Serum blood glucose, mg/dL	−22.588 (−39.531, −12.209)	<0.001
C peptide, nmol/liter	0.053 (0.042, 0.064)	<0.001
Hemoglobin A1c, %	−1.01 (−1.251, −0.768)	<0.001
Initial insulin dose, U/kg/d	−0.19 (−0.219, −0.170)	<0.001

Logistic and linear regression models were adjusted for age at diagnosis, sex, and parental consanguinity. * Adjusted for gender and consanguinity.

## Data Availability

All the necessary information is provided within the manuscript. Any other data that support the findings of this study are available from the first author upon request.
